# Silk Fibroin-Alginate-Hydroxyapatite Composite Particles in Bone Tissue Engineering Applications In Vivo

**DOI:** 10.3390/ijms18040858

**Published:** 2017-04-18

**Authors:** You-Young Jo, Seong-Gon Kim, Kwang-Jun Kwon, HaeYong Kweon, Weon-Sik Chae, Won-Geun Yang, Eun-Young Lee, Hyun Seok

**Affiliations:** 1 Sericultural & Apicultural Materials Division, National Institute of Agricultural Science, Wanju 55365, Korea; yyjo@korea.kr (Y.-Y.J.); hykweon@korea.kr (H.K.); 2 Department of Oral and Maxillofacial Surgery, Gangneung-Wonju National University, Gangneung 25457, Korea; epker@chol.com (S.-G.K.); jf1225@gwnu.ac.kr (K.-J.K.); 3 Analysis Research Division, Daegu Center, Korea Basic Science Institute, Daegu 41566, Korea; Wschae@kbsi.re.kr (W.-S.C.); wgyang1@kbsi.re.kr (W.-G.Y.); 4 Department of Oral and Maxillofacial Surgery, Chungbuk National University College of Medicine, Cheongju 28644, Korea; ley926@chungbuk.ac.kr; 5 Department of Oral and Maxillofacial Surgery, Chungbuk National University Hospital, Cheongju 28644, Korea

**Keywords:** silk fibroin, alginate, hydroxyapatite, tumor necrosis factor α

## Abstract

The aim of this study was to evaluate the in vivo bone regeneration capability of alginate (AL), AL/hydroxyapatite (HA), and AL/HA/silk fibroin (SF) composites. Forty Sprague Dawley rats were used for the animal experiments. Central calvarial bone (diameter: 8.0 mm) defects were grafted with AL, AL/HA, or AL/HA/SF. New bone formation was evaluated by histomorphometric analysis. To demonstrate the immunocompatibility of each group, the level of tumor necrosis factor (TNF)-α expression was studied by immunohistochemistry (IHC) and quantitative reverse transcription polymerase chain reaction (qRT-PCR) at eight weeks post implantation. Additionally, osteogenic markers, such as fibroblast growth factor (FGF)-23, osteoprotegerin (OPG), and Runt-related transcription factor (Runx2) were evaluated by qPCR or IHC at eight weeks post implantation. The AL/HA/SF group showed significantly higher new bone formation than did the control group (*p* = 0.044) and the AL group (*p* = 0.035) at four weeks post implantation. Additionally, the AL/HA/SF group showed lower relative TNF-α mRNA levels and higher FGF-23 mRNA levels than the other groups did at eight weeks post implantation. IHC results demonstrated that the AL/HA/SF group had lower TNF-α expression and higher OPG and Runx2 expression at eight weeks post implantation. Additionally, no evidence of the inflammatory reaction or giant cell formation was observed around the residual graft material. We concluded that the AL/HA/SF composite could be effective as a scaffold for bone tissue engineering.

## 1. Introduction

Bone is an essential organ of the vertebrate skeleton [1]. It protects and provides support for various organs of the body [2]. Bone is a natural composite comprising both organic and inorganic components [3]. In bone, inorganic biominerals are embedded in an organic matrix consisting mainly of collagen proteins [4]. Moreover, 70 wt % of bone is composed of an inorganic matrix formed mainly from hydroxyapatite (HA) [4], a thermodynamically stable calcium-phosphate salt with the molecular formula Ca_10_(OH)_2_(PO_4_)_6_ [5]. The inorganic biominerals of bone are chemically similar to those of HA [6]. This chemical similarity has led to the extensive application of HA in bone tissue engineering [3]. HA has excellent biocompatibility and bioactivity with less toxicity and inflammation [3]. Additionally, HA has osteoconductive properties and osteoinductive potential as a bone graft material [7]. HA has been made from various sources, including eggshells, coral, and demineralized bovine or human bones [3]. It has also been widely used as a component of bone substitute material by blending with various polymers, including collagen, polycaprolactone, chitosan, alginate (AL), and silk fibroin (SF) [8].

The biomedical applications of AL, a natural anionic polymer obtained from brown seaweed, have been widely investigated due to the biocompatibility, low toxicity, gelation capacity, and cost effectiveness of AL [9]. Indeed, AL can be easily modified into different forms of matrices, including microcapsules, spheres, hydrogels, fibers, and foams [10]. Therefore, AL is used with different polymers for production of tissue engineering scaffolds and delivery vehicles for cells, growth factors, or drugs [9]. Bead-structured matrices are easily prepared by manipulating AL gelation in the presence of divalent ions, such as Ca^2+^ [11]. Such divalent ions bind and crosslink AL polymer chains [11]. AL gelatin is formed by the crosslinking of each AL polymer [11]. The drawback of AL is poor mechanical strength in the load-bearing area [12]. Though chemical modification may improve the mechanical properties of AL, these reactions decrease biocompatibility [13]. For bone graft scaffolds, AL is used as a blended composite in combination with calcium phosphate composites, such as HA and biphasic calcium phosphate (BCP) [14].

SF is a commonly available natural biopolymer produced by silkworms [15]. Silk fiber from *Bombyx mori* is composed of fibroin and sericin [16]. To prevent the immune reaction, the sericin is removed by degumming, and the resulting SF is used as a tissue engineering scaffold [17,18]. The application of SF for tissue engineering has been widely explored, as SF can be easily processed in aqueous solution and has desirable properties, including mechanical strength, biocompatibility, nontoxicity, biodegradability, and permeability [15]. SF from the mulberry silk worm *B. mori* is composed of heavy (H) and light (L) chains linked with disulfide bonds and P25, a 25-kDa protein noncovalently linked to these chains [19]. The ratio of H-fibroin, L-fibroin, and P25 is 6:6:1 in silk from *B. mori* [20]. The use of an electrospun SF matrix has been shown to improve the mechanical strength of a 20 wt % HA reinforcement [21]. Surface coating, through biomineralization of an electrospun SF matrix, enhances cell growth and bone regeneration [22]. An HA coating on the SF scaffolds enhances osteoconductivity and osteoinductivity [23]. Therefore, incorporation of HA into SF matrices is expected to form materials that are useful for bone tissue engineering applications.

Implantation foreign body scaffolds into a host can produce an immune reaction and trigger the secretion of inflammatory cytokines [24]. Pro-inflammatory cytokines, such as interleukin (IL)-2, IL-6, and tumor necrosis factor α (TNF-α), are closely related to inflammation-induced bone resorption [25]. In particular, the expression of TNF-α in bone defect areas can disrupt and impair bone regeneration [26]. TNF-α, an inflammatory mediator, is produced by macrophages and many other cells, including CD^4+^ lymphocytes, neutrophils, and mast cells [27]. TNF-α is associated with systemic inflammatory reactions and expressed in the acute inflammatory phase [26]. The regulation of TNF-α expression is an important therapeutic target for successfully grafting bone in patients with autoimmune disease [28,29]. Several markers have been shown to indicate bone regeneration, including fibroblast growth factor-23 (FGF-23), osteoprotegerin (OPG), and Runt-related transcription factor 2 (Runx2) [30,31,32].

AL/SF composites have been introduced as biomaterial scaffolds for soft tissue and osteochondral tissue engineering [33]. However, few studies have reported the applications of AL/SF composites as bone tissue regeneration scaffolds. AL combined with HA exhibits excellent osteoinductive and osteogenic activity in vitro [34]. Additionally, AL-gelatin-BCP hydrogels containing HA show significantly higher bone formation in vivo [14]. However, the application of AL/HA/SF composites as bone tissue engineering scaffolds has rarely been reported.

Therefore, in this study, we aimed to evaluate the bone regeneration efficacy of the AL/HA/SF composite in vivo by analyzing TNF-α, FGF-23, OPG, and Runx2 expression levels. We prepared AL/HA/SF particles and characterized their effects on bone tissue regeneration using an animal model. New bone formation stimulated by AL/HA/SF was evaluated by measuring the sizes of rat calvarial defects, and the expression levels of TNF-α, FGF-23, OPG, and Runx2 were evaluated in each group. The results provided important insights into the appropriate biomaterials for bone tissue engineering.

## 2. Results

### 2.1. Morphology ofAlginate (AL), AL/Hydroxyapatite (HA), and AL/HA/Silk Fibroin (SF) Particles in Scanning Electron Microscope (SEM) Images

**Figure 1:**
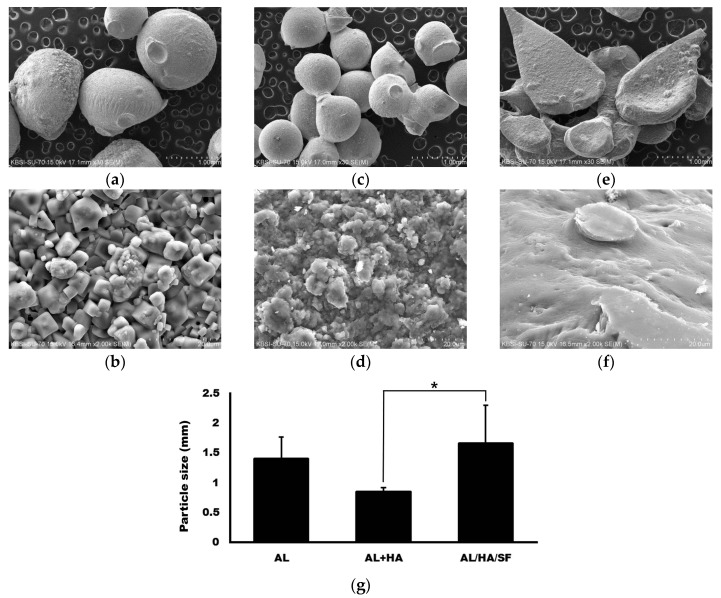
Scanning microscopic images. Alginate (AL) particles ((**a**,**b**); original magnification 30×, 2000, respectively), AL/ Hydroxyapatite (HA) composites ((**c**,**d**); original magnification 30×, 2000×, respectively), AL/HA/ Silk fibroin (SF) composites ((**e**,**f**); original magnification 30×, 2000×, respectively); (**g**) There was a significant difference between the AL/HA and AL/HA/SF groups with regard to particle size (* *p* = 0.003).

SEM images of AL, AL/HA, and AL/HA/SF particles are presented in Figure 1. Particles of AL and AL/HA were spherical (Figure 1). However, particles in the AL/HA/SF group had a flattened teardrop shape. Mean particle sizes were 1.41 ± 0.36, 0.85 ± 0.06, and 1.65 ± 0.65 mm in the AL, AL/HA, and AL/HA/SF groups, respectively (Figure 1g). The differences among groups were statistically significant (*p* = 0.004). In post hoc tests, the difference between the AL/HA group and the AL/HA/SF group was statistically significant (*p* = 0.003). However, the differences between the other groups were not statistically significant (*p* > 0.05). The AL particles had rough surfaces, whereas the surfaces of the AL/HA particles were even rougher due to HA components (Figure 1c,d). The AL/HA/SF particles had smooth surfaces and formed aggregates because SF attached to the surface of the particles (Figure 1e,f).

### 2.2. Attenuated Total Reflection Fourier Transform Infrared (ATR-FT-IR) Spectroscopy

**Figure 2:**
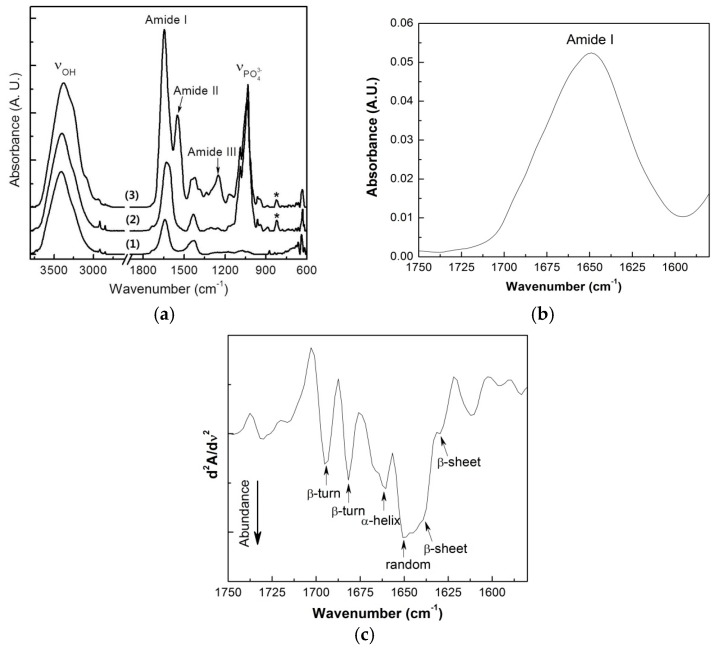
(**a**) Attenuated Total Reflection Fourier Transform Infrared (ATR-FT-IR) absorption spectra of the (**1**) Alginate (AL), (**2**) AL/Hydroxyapatite (HA), and (**3**) AL/HA/Silk fibroin (SF) beads (* indicates the absorption band of HA composition); (**b**,**c**) the infrared absorption and second derivative spectrum of the amide I of the composited SF.

Figure 2 shows the infrared absorption for AL and the HA and SF composite forms. The AL base material showed strong and broad absorption at 3000–3700 cm^−1^ due to the stretching vibrations of the abundant hydrogen-bonded O–H groups. The weak peaks at 2918 and 2850 cm^−1^ represented aliphatic C–H stretching. The infrared absorption peaks appearing at 1641 and 1428 cm^−1^ could be assigned to C=O vibrations and C–OH deformation vibrations, respectively [35]. In the HA composite form, typical vibrational absorption for HA was clearly observed at 1034 and 1089 cm^−1^, originating from the asymmetric stretching of PO_4_^3−^, and the corresponding symmetric stretching peak was present at 823 cm^−1^ (indicated by asterisks) [36,37].

When the AL/HA was treated with SF, several strong vibrational peaks appeared in the 1200–1700 cm^−1^ region. These peaks corresponded to typical vibrational absorption of protein secondary structures. The absorption bands at 1645 and 1550 cm^−1^ could be attributed to amide I (C=O stretching) and amide II (C–N stretching and N–H bending), respectively. In particular, the amide I peak position is known to be very sensitive to the protein secondary structure; thus, this peak was frequently used to analyze protein structure. However, the broad absorption peaks made it difficult to distinguish the subcomponent absorption for each protein secondary structure. To solve this problem, researchers previously suggested that the second derivative spectrum of the infrared amide I peak could clearly separate the subcomponent peaks [38]. Figure 2b,c shows the amide I peak and the second derivative infrared absorption spectrum of the SF. The second derivative spectrum clearly revealed that the SF had secondary protein structures consisting of random coils (1650 cm^−1^), β-sheets (1631 and 1639 cm^−1^), α-helixes (1660 cm^−1^), and β-turns (1682 and 1695 cm^−1^). Random coil and β-sheet conformations were major components of the SF-containing composite, and the α-helix and β-turn conformations were present as minor components. The absorption band at 1250 cm^−1^ was attributed to amide III, also indicating the presence of a random coil conformation.

### 2.3. Analysis of Cytotoxicity

**Figure 3:**
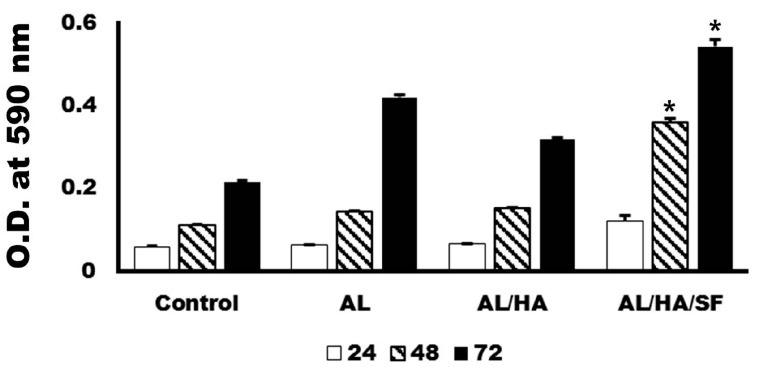
3-(4,5-Dimethylthiazol-2-Yl)-2,5-diphenyltetrazolium bromide (MTT) assays for MG63 cells on each particle (AL, AL/HA, or AL/HA/SF) at 24, 48, and 72 h of culture. Cell growth in AL/HA/SF groups was significantly faster than that in the AL/HA group at 48 and 72 h (O.D.: optic density, * *p* < 0.001).

Cells loaded on each particle were quantitatively assessed for cellular metabolic activity with 3-(4,5-Dimethylthiazol-2-Yl)-2,5-diphenyltetrazolium bromide (MTT) after 24, 48, and 72 h of culture. The cells grown on each particle demonstrated good cellular metabolic activity throughout the culture period. The MTT assay results indicated that the growth of MG63 cells at 48 and 72 h was faster in the AL/HA/SF group than in the AL/HA group (Figure 3; *p* < 0.001).

### 2.4. Histologic and Histomorphometric Evaluation Using Hematoxylin and Eosin Staining

**Figure 4:**
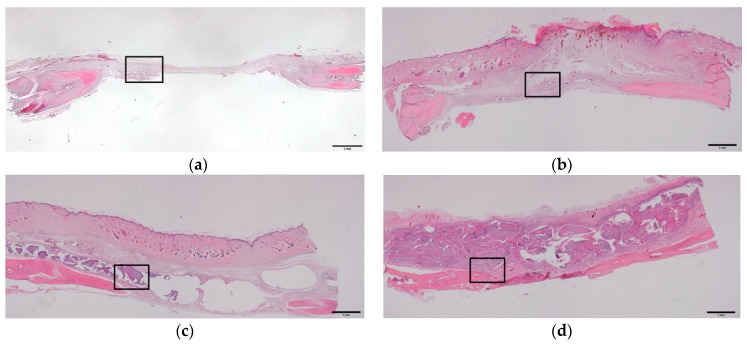

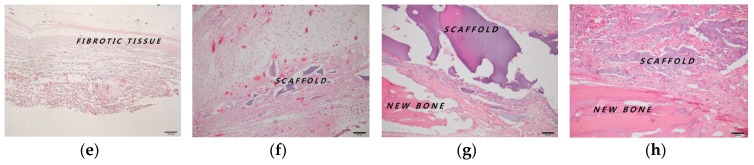
Histological images (hematoxylin and eosin staining) at four weeks after operation. (**a**) unfilled defects from the control group (bar = 1 mm); (**b**) AL (bar = 1 mm); (**c**) AL/HA (bar = 1 mm); and (**d**) AL/HA/SF (bar = 1 mm) beads; (**e**–**h**) magnified views of the boxed areas in (**a**–**d**) (original magnification 100×, bar = 100 µm).

The histomorphometric evaluation of each group is presented in Table 1. New bone formation rates at four weeks after operation in the control, AL, AL/HA, and AL/HA/SF groups were 13.87 ± 4.98%, 14.32 ± 6.39%, 28.60 ± 12.57%, and 30.93 ± 11.05%, respectively. New bone formation in the AL/HA/SF group was significantly higher than those in the control group (*p* = 0.044) and AL group (*p* = 0.035). The residual graft rates were 25.38 ± 13.55% in the AL group, 31.85 ± 9.98% in the AL/HA group, and 33.93 ± 16.45% in the AL/HA/SF group at four weeks after the operation (Figure 4f–h). These differences were not significant (*p* > 0.05). In the AL/HA/SF group, low inflammation and a few giant cells were observed around the grafted material (Figure 4f–h).

**Figure 5:**
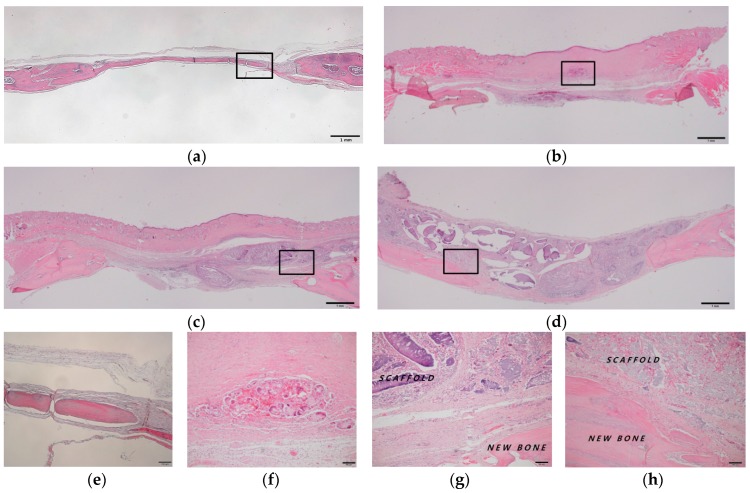
Histological images (hematoxylin and eosin staining) at eight weeks after operation. (**a**) unfilled defect in the control group (bar = 1 mm). (**b**) AL (bar = 1 mm), (**c**) AL/HA (bar = 1 mm), (**d**) and AL/HA/SF (bar = 1 mm) composites. (**e**–**h**) are magnified views of the boxed areas in (**a**–**d**) (original magnification 100×, bar = 100 µm).

New bone formation rates at eight weeks after operation in the control, AL, AL/HA, and AL/HA/SF groups were 23.85 ± 2.21%, 27.00 ± 11.59%, 33.25 ± 5.69%, and 39.46 ± 12.92%, respectively. The average of total new bone formation in the AL/HA/SF group was higher than that of other groups. However, there were no significant differences among groups. The residual graft rates were 20.75 ± 11.70% in the AL group, 29.25 ± 8.89% in the AL/HA group, and 25.12 ± 0.83% in the AL/HA/SF group at eight weeks after the operation (Figure 5f–h). These differences were not significant (*p* > 0.05). The AL group showed low inflammation and giant cell formation around the grafted material (Figure 5f). Degradation of the grafted material was observed in the AL/HA and AL/HA/SF groups (Figure 5g,h).

### 2.5. Analysis of TNF-α Expression

**Figure 6:**
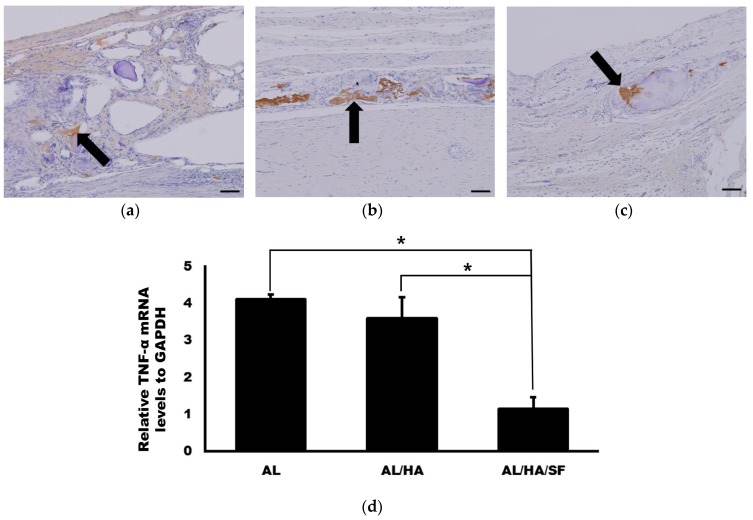
Immunohistochemical staining for tumor necrosis factor-α (TNF-α) (original magnification, 200×, bar = 50 µm) and qRT-PCR of TNF-α mRNA at 8 weeks after operation. TNF-α positive area was indicated as arrows. (**a**) AL group; (**b**) AL/HA group; (**c**) AL/HA/SF group; (**d**) Significantly lower relative TNF-α expression was observed in the AL/HA/SF group compared with that in the AL and AL/HA groups (* *p* < 0.001 and *p* = 0.001, respectively).

The expression level of TNF-α was evaluated at eight weeks after operation. TNF-α expression detected by immunohistochemistry (IHC) is presented in Figure 6. TNF-α expression was broadly distributed in the AL group. However, TNF-α expression was specifically localized in the AL/HA and AL/HA/SF groups. The expression levels of TGF-α relative to those in the control group were 4.11 ± 0.16, 3.60 ± 0.56, and 1.15 ± 0.72 in the AL, AL/HA, and AL/HA/SF group, respectively (Figure 6d). The expression of TGF-α was significantly lower in the AL/HA/SF group than in the AL and AL/HA groups (*p* < 0.001 and *p* = 0.001, respectively).

### 2.6. Analysis of Osteogenic Markers

**Figure 7:**
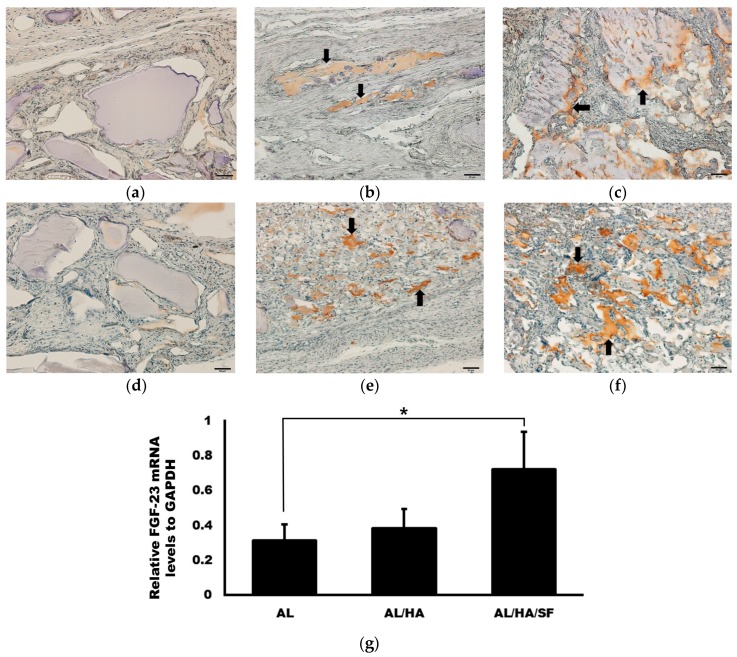
Immunohistochemical staining for osteoprotegerin (OPG) (**a**–**c**) and Runx2 (**d**–**f**, original magnification, 200×, bar = 50 µm) and qRT-PCR of FGF-23 mRNA at 8 weeks after operation. TNF-α positive area was indicated as arrows. (**a**,**d**) AL group; (**b**,**d**) AL/HA group; (**c**,**f**) AL/HA/SF group; (**g**) Significantly higher FGF-23 expression was observed in the AL/HA/SF group compared with that in the AL group (* *p* = 0.044, respectively).

The expression levels of OPG and Runx2 were evaluated at eight weeks after operation. OPG and Runx2 expression detected by IHC is presented in Figure 7. OPG and Runx2 expression was higher in the AL/HA/SF group. However, low levels of OPG and Runx2 expression were observed in the AL group. The expression levels of FGF-23 relative to that in the control group were 0.31 ± 0.09, 0.39 ± 0.11, and 0.72 ± 0.21 for the AL, AL/HA, and AL/HA/SF groups, respectively. The relative expression of FGF-23 in the AL/HA/SF group was significantly higher than that in the AL group (*p* = 0.044).

## 3. Discussion

In this study, two different types of AL composites were compared with AL scaffolds. Different composites had different sizes and morphologies (Figure 1). FT-IR spectra demonstrated successful integration of HA or SF into the blended composite (Figure 2). MTT assays demonstrated that the growth of MG63 cells at 48 and 72 h was faster in the AL/HA/SF group than in the AL/HA group (Figure 3; *p* < 0.001). In an animal study, the AL/HA/SF group showed less TNF-α expression than the other groups (Figure 6). Additionally, elevated expression levels of osteogenic markers, such as FGF-23, OPG, and Runx2, were found in the AL/HA/SF group (Figure 7). Consequently, new bone formation was the highest in the AL/HA/SF group among the three tested groups (Table 1).

The AL, AL/HA, and AL/HA/SF particles were spherical with rough surfaces as shown by SEM images (Figure 1). AL/HA/SF particles formed aggregates due to their coating of SF material and it shows flattened and teardrop shape compared with other particles (Figure 1e). Fine HA granules were observed on the surfaces of AL/HA particles (Figure 1c). Differences in particle sizes between the AL/HA and AL/HA/SF groups were found to be statistically significant (*p* = 0.003). Furthermore, there is more size distribution in AL/HA/SF than other groups. Because of the bigger size and irregular structure, AL/HA/SF particles had larger area to contract with osteogenic cells. SF has a positive effect on osteoblast-like cell adhesion and proliferation [39]. The structure of AL/HA/SF particles may influence osteogenic cell proliferation and bone regeneration. In a previous clinical trial, researchers found that the particle size of the graft did not influence the success of dental implants in maxillary sinus applications [40]. However, another study demonstrated that highly porous medium sized particles showed the best performance as bone graft materials [41]. Because the influence of graft size and structure on bone regeneration is controversial, further studies are required to assess the effects of size and structure on successful bone grafting. In ATR-FT-IR spectroscopy, all components showed successful blending into composites (Figure 2). The AL, AL/HA, and AL/HA/SF particles showed different chemical composition, size and shape.

The development of blended composites is aimed at identifying the best composition to compensate for the drawbacks of materials containing only a single component. In this study, both the AL group and the AL/HA/SF group showed higher values in MTT assays than the AL/HA group (Figure 3). Porous AL scaffolds have been shown to promote excellent MG-63 cell adhesion and proliferation in vitro and to enhance bone regeneration and osteogenesis in vivo [42]. Moreover, SF/HA composite coating yields higher values in MTT assays than HA coating alone in MG-63 cell cultures [43]. SF has been shown to stimulate osteogenesis by increasing the expression of osteogenic genes in MG-63 cells [44]. Hence, SF may contribute to the proliferation of osteogenic cells and regeneration of new bone. In this study, we prepared AL beads combined with SF and HA and evaluated the bone regeneration efficacy of this material when used as a bone tissue engineering scaffold. 

At four weeks after the operation, new bone formation in the AL/HA/SF group was 30.93 ± 11.05%, which was significantly higher than those in the control group (13.87 ± 4.98%, *p* = 0.044) and the AL group (14.32 ± 6.39%, *p* = 0.035). At eight weeks after the operation, average new bone formation in the AL/HA/SF group was higher than that in the AL and AL/HA groups (*p* > 0.05). SF has been shown to create a more osteogenic microenvironment by enhancing osteogenic cell differentiation within bone defects [39,45]. Additionally, SF has been shown to stimulate the expression of osteogenic genes, such as alkaline phosphatase (ALP), osteocalcin, and osterix, thus promoting osteoblastic differentiation [46]. In a previous in vivo study, SF scaffolds were used as the main component of a bone substitute and were found to stimulate bone regeneration when implanted into rat calvarial defects [47]. Additionally, SF membranes cause accelerated healing of bone defects using the guided bone regeneration technique [48,49]. Because of these properties of SF, we speculated that SF could contribute to new bone regeneration in calvarial bone defects without inducing inflammatory reactions.

SF composites have been shown to have excellent bone regeneration capability and potential as bone tissue engineering scaffolds in previous in vivo and in vitro studies [46,47]. Generally, SF material is considered biocompatible and has little immunogenic activity [40]. To prevent an inflammatory reaction, silk is processed through a degumming procedure for the removal of sericin [17,18]. At the histological level, the AL/HA/SF group exhibited no inflammatory reaction or giant cell formation around the area of the residual graft material (Figure 4h and Figure 5h). A mild inflammatory reaction and granulomatous tissue formation were observed only in the AL group (Figure 5f). Furthermore, the residual grafted material in the AL/HA/SF group (25.12 ± 0.83%) was lower than that in the AL/HA group (29.25 ± 8.89%) at eight weeks after operation. However, there was no significant difference in the amount of residual graft material (*p* > 0.05) among the AL, AL/HA, and AL/HA/SF groups. These results demonstrated that the SF had no effect on the biodegradation of the composite material or on the foreign body inflammatory reaction.

The success of SF scaffold implantation is closely related to TNF-α expression [28,50]. TNF-α is a pro-inflammatory cytokine that can be produced by macrophages during biomaterial degradation [51]. TNF-α has been shown to inhibit the differentiation of osteogenic cells and osteogenesis of mesenchymal stem cells [52]. Moreover, TNF-α has also been shown to stimulate inflammation-driven bone resorption and negatively affect new bone formation [53]. TNF-α is associated with osteoclastogenesis and enhances the differentiation of osteoclasts via receptor activator of nuclear factor-κB ligand (RANKL) signaling [54,55]. Immunohistochemistry revealed that TNF-α expression was increased in the AL group (Figure 6). Additionally, the relative level of TNF-α mRNA in the AL/HA/SF group was significantly lower than those in the AL and AL/HA groups (Figure 6d; *p* < 0.001 and *p* = 0.001, respectively). AL is known to induce secretion of TNF-α from macrophages [56]. Therefore, elevated levels of TNF-α may be related to the presence of AL. This result also indicated that the SF did not trigger TNF-α secretion and induced a host immunological reaction. This conclusion was also supported by histological analysis of the AL/HA/SF group, which showed no evidence of giant cell formation or the inflammatory reaction. In a previous study, SF material did not promote TNF-α secretion in comparison with that of other biomaterials [57]. Although several reports have demonstrated that SF scaffolds induce TNF-α secretion as early as one week after implantation, the TNF-α levels return to baseline 2–4 weeks later [28,51]. Additionally, in another in vivo study, the SF base material was found to effectively inhibit the transcription of pro-inflammatory cytokines, including IL-1β, IL-6, and TNF-α [50]. TNF-α is associated with the inhibition of osteogenic genes, including bone morphogenic protein 2, osteocalcin, and Runx2 [58]. From the results of our study, we confirmed that AL/HA/SF scaffolds caused minimal inflammation and did not induce TNF-α expression. Consequently, AL/HA/SF scaffolds promote new bone formation without a foreign body reaction.

In IHC analysis, elevation of OPG and Runx2 expression was observed in the AL/HA/SF group (Figure 7). OPG and Runx2 are closed related to bone development and regeneration [30,31,32]. Runx2 is a major transcription factor involved in osteogenesis [31] and is essential for bone development and osteoblast differentiation from mesenchymal cells [59]. OPG produced by osteoblasts and osteocytes inhibits osteoclast differentiation and activation [32]. Moreover, OPG also regulates bone remodeling and inhibits excessive bone resorption by binding the RANKL [60]. Significantly higher expression of FGF-23 was found in the AL/HA/SF group than in the AL group (Figure 7g, *p* = 0.044). FGF-23 regulates phosphate homeostasis in serum and modulates vitamin D metabolism [61]. Additionally, FGF-23 is primarily secreted in bone tissues during osteoblast development [61]. In IHC analysis, the expression of OPG and Runx2 was not found in the AL group. However, higher expression of OPG and Runx2 was observed in the AL/HA/SF group, consistent with the observed increase in bone formation in the AL/HA/SF group. Elevation of FGF-23 in the AL/HA/SF group also demonstrated the osteogenic properties of SF. Thus, our results showing that osteogenic markers were elevated supported the bone regeneration capacity of AL/HA/SF particles.

## 4. Materials and Methods

### 4.1. Preparation of AL, AL/HA, and AL/HA/SF Composites

*B. mori* cocoons were used for generating silk solutions. Alginic acid sodium salt from brown algae (Sigma-Aldrich, St. Louis, MO, USA) and HA were purchased for this study. The preparation procedures for AL, AL/HA, and AL/HA/SF were described in previous publications [62,63]. Briefly, Mulberry silk cocoons were cut and degummed in 0.2 M Na_2_CO_3_ for 30 min followed by drying at 37 °C for 24 h. Degummed fibers were dissolved in 9.3 M LiBr and incubated for 4 h at 60 °C. The protein solution was dialyzed against deionized water using a 12-kDa membrane for 72 h. The concentration of fibroin was determined to be 3% (*w*/*v*). A sodium AL solution (3% *w*/*v*) was prepared in 1× phosphate-buffered saline (PBS). A measured amount of HA was dispersed in a small volume of 1× PBS by ultrasonication for 30 min. The final concentration of prepared mixing AL, fibroin, and dispersed HA solution was 1.5% *w*/*v*, 1% *w*/*v*, and 30% *w*/*w*, respectively. The prepared solutions were added drop by drop (5 mL/min) through a microtip nozzle into a 0.2 M CaCl_2_ solution. The tip end was 3 cm above the CaCl_2_ solution. Composites were aged for 12 h for complete ion exchange and dried at 37 °C.

### 4.2. Scanning Electron Microscopy (SEM)

AL, AL/HA and AL/HA/SF beads were examined by SEM using an electron microscope (SU-70; Hitachi, City, Japan) at 5 keV at the KSBI Gangneung center. The maximum length of the particles was measured using an image analysis program with the SEM images.

### 4.3. ATR-FT-IR Spectroscopy

ATR-FT-IR absorption spectra of the AL and AL/HA/SF composite were measured using a Fourier transform spectrometer (Vertex 80; Bruker Optics, Bremen, Germany) equipped with an attenuated total reflectance accessory (MIRacle; PIKE Technologies, Fitchburg, WI, USA). Spectra were recorded in the range of 600–4000 cm^−1^ at a resolution of 2 cm^−1^ with a DLaTGS detector (Bruker Optics). One hundred twenty-eight repeated scans were averaged for each spectrum.

### 4.4. MTT Assay

In vitro cell cultures were performed using MG63 osteoblast-like cells (ATCC, Manassas, VA, USA). The cells were grown to 80% confluence in α-minimum essential media (MEM) (HyClone, Logan, UT, USA) and Dulbecco’s modified Eagle’s medium-high glucose (PAA Laboratories, Linz, Austria) containing 1% penicillin/streptomycin (100×) supplemented with 10% autologous serum. The cells were kept at 37 °C in an atmosphere of 5% CO_2_ and 99% relative humidity. Each particle (AL, AL/HA, or AL/HA/SF) was placed in the 24-well plate and washed with cell culture medium. A total of 5 × 10^4^ cells were seeded into each well on the particle, and the viability of MG63 cells was assessed after 24, 48, and 72 h of growth on each particle type (AL, AL/HA, or AL/HA/SF) using 3-(4,5-dimethylthiazole-2-yl)-2,5-diphenyltetrazolium bromide (MTT) assays. Cell culture without particles was used as a control. Subsequent procedures were performed according to the manufacturer’s protocol (Cell Proliferation Kit I; Roche Molecular Biochemicals, Mannheim, Germany). The results were estimated by measuring the absorbance at 590 nm with a Victor Multilabel Counter (Perkin-Elmer-Wallac, Freiburg, Germany).

### 4.5. Animal Experiments

Forty 12-week-old Sprague Dawley rats were used in this study. The average weight of the rats was 300 g. This study was approved by the Institutional Animal Care and Use Committee of the Gangneung-Wonju National University, Gangneung, Korea (IACUC GWNU-2015-9). 

**Figure 8:**
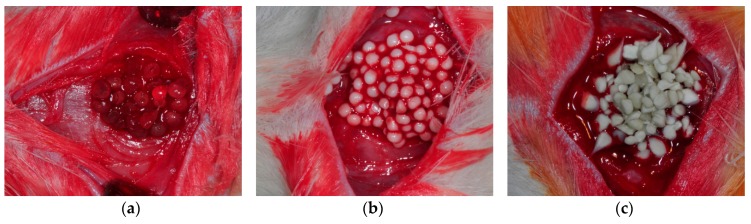
(**a**) Alginate; (**b**) HA/alginate; and (**c**) SF/HA/alginate beads were grafted into rat calvarial defects.

An intramuscular injection of a combination of Zoletil 50 (15 mg/kg; Vibac, Carros, France) and Rumpun (0.2 mL/kg; Bayer Korea, Seoul, Korea) was performed for general anesthesia. The cranial area of the skull was shaved and then disinfected with povidone-iodine. Local anesthesia was performed on the cranial area with an injection of 2% lidocaine with epinephrine (1:100,000). A longitudinal incision was made in the midsagittal plane. Sharp subperiosteal dissection was performed to reflect the pericranium and expose the calvarial bone. The bone defect was created using a trephine bur with saline irrigation. The defect was 8 mm in diameter and 2 mm in depth. Calvarial defects were grafted with AL, AL/HA and AL/HA/SF beads (Figure 8). Some defects were left empty for the control group. After grafting, the muscle and skin were closed with 3–0 black silk (AILEE, Busan, Korea). Gentamycin (1 mg/kg; Kookje, Seoul, Korea) and pyrin (0.5 mL/kg; Green Cross Veterinary Products, Seoul, Korea) were injected intramuscularly three times daily for 3 days. All rats were housed separately and received food and water ad libitum. Five rats from each group were sacrificed at 4 and 8 weeks after surgery. Specimens were fixed in 10% formalin. Histological analysis was performed to evaluate new bone formation.

### 4.6. Histologic and Histomorphometric Evaluation

At 4 and 8 weeks after surgery, the rats were sacrificed and the calvarial bone defects were harvested. All specimens were decalcified in 5% nitric acid for 2 weeks and dehydrated in ethyl alcohol and xylene. The specimens were embedded in paraffin blocks and sectioned prior to staining with hematoxylin and eosin. Images of the stained slices were captured with a digital camera (DP-73; Olympus, Tokyo, Japan). Digital images of selected sections were analyzed by Sigma Scan pro (SPSS, Chicago, IL, USA). The amount of new bone formation is displayed as the percentage of the total bone defect region. The area of residual graft material is displayed as a percentage of the original area of the graft material.

### 4.7. Immunohistochemical Evaluation of TNF-α Expression

IHC was performed on histological sections to evaluate the expression of TNF-α, OPG, and Runx2. Anti-TNF-α (Santa Cruz Biotechnology, Santa Cruz, CA, USA), anti-OPG (sc-8468; Santa Cruz Biotechnology), and anti-Runx2 (sc-10758; Santa Cruz Biotechnology) antibodies were used as primary antibodies. Immunohistochemical staining was performed using a Dako REAL EnVision Detection System (Dako, Glostrup, Denmark) according to the manufacturer’s protocols. Counterstaining was conducted with Mayer’s hematoxylin (Sigma-Aldrich). Stained tissue slides were examined with an Olympus BX51 (Olympus, Tokyo, Japan) microscope.

### 4.8. qRT-PCR

RNA was extracted from paraffin sections using a MagMAX FFPE DNA/RNA Ultra kit (Thermo Fisher Scientific, Austin, TX, USA). For RNA extraction, tissues were cut to 50 µm thickness. After removing paraffin, tissue pellets were collected and washed with ethanol. Subsequent procedures were performed in accordance with the manufacturer’s instructions. Extracted RNA was reverse-transcribed, and cDNA was used as a template for qRT-PCR. Primers for FGF-23 and TNF-α were designed based on previous publications [61,64]. Subsequent procedures for qRT-PCR were performed in accordance with our previous publication [3]. The negative control was a mixture without template. The relative expression of each gene is shown as the ratio of the target gene expression to the expression of glyceraldehyde 3-phosphate dehydrogenase (GAPDH). The average value of each group was used for comparison.

### 4.9. Statistical Analysis

One-way analysis of variance (ANOVA) was used for comparisons of three or more independent groups. Bonferroni's method was used for post hoc tests. Differences with *p*-values of less than 0.05 were considered significant.

## 5. Conclusions

In this study, we evaluated AL/HA/SF composites as a bone tissue regeneration scaffold. At four weeks after operation, the AL/HA/SF group showed significantly higher new bone formation compared with the control and AL groups (*p* = 0.044 and *p* = 0.035, respectively). Additionally, the residual graft material in the AL/HA/SF group was lower than that in the AL/HA group at eight weeks after operation. Because of the biocompatibility of AL/HA/SF, the scaffolds did not induce an inflammatory reaction or giant cell formation around the residual graft material. The AL/HA/SF group showed significantly lower expression of TNF-α compared with the AL and AL/HA groups (*p* < 0.001 and *p* = 0.001, respectively). Moreover, high expression of osteogenic markers, such as FGF-23, OPG, and Runx2, was observed in the AL/HA/SF group. From these results, we concluded that the AL/HA/SF scaffolds contributed to new bone regeneration in rat calvarial defects and were stably biodegraded without inducing foreign body inflammatory reactions. We propose that AL/HA/SF can be effective scaffolds for bone tissue engineering applications.

## Figures and Tables

**Table 1:** Histomorphometric analysis.

Post-Implantation	4 Weeks			
Group	Control	AL	AL/HA	AL/HA/SF
Total new bone (%)	13.87 ± 4.98	14.32 ± 6.39	28.60 ± 12.57	30.93 ± 11.05
Residual graft (%)		25.38 ± 13.55	31.85 ± 9.98	33.93 ± 16.45
**Post-Implantation**	**8 Weeks**			
Group	Control	AL	AL/HA	AL/HA/SF
Total new bone (%)	23.85 ± 2.21	27.00 ± 11.59	33.25 ± 5.69	39.46 ± 12.92
Residual graft (%)		20.75 ± 11.70	29.25 ± 8.89	25.12 ± 0.83

AL: alginate, HA: hydroxyapatite, SF: silk fibroin.
